# MCP-1/MCPIP-1 Signaling Modulates the Effects of IL-1β in Renal Cell Carcinoma through ER Stress-Mediated Apoptosis

**DOI:** 10.3390/ijms20236101

**Published:** 2019-12-03

**Authors:** Chia-Huei Lee, Pin-Feng Hung, Shang-Chieh Lu, Hsuan-Lien Chung, Shang-Lun Chiang, Chun-Te Wu, Wei-Chun Chou, Chiao-Yin Sun

**Affiliations:** 1 National Institute of Cancer Research, National Health Research Institutes, Zhunan 35053, Taiwan; hdp91111@nhri.org.tw (P.-F.H.); zzz810355@nhri.edu.tw (H.-L.C.); 2 Department of Nephrology, Chang Gung Memorial Hospital, Keelung 20401, Taiwan; lsjstella0808@gmail.com; 3 Department of Health Risk Management, College of Public Health, China Medical University, Taichung 40402, Taiwan; chimpanzee99999@gmail.com; 4 Department of Urology, Chang Gung Memorial Hospital, Keelung 20401, Taiwan; chuntewu@cgmh.org.tw; 5 Department of Medical Research, School of Medicine, Chung Gung University, Taoyuan 33302, Taiwan; 6 Department of Anatomy and Physiology, Institute of Computational Comparative Medicine, College of Veterinary Medicine, Kansas State University, Manhattan, KS 66506, USA; weichunc@vet.k-state.edu

**Keywords:** interleukin-1β, chemoattractant protein-1, renal cell carcinoma, monocyte chemoattractant protein (MCP)-induced protein-1, endoplasmic reticulum stress, apoptosis

## Abstract

In renal cell carcinoma (RCC), interleukin (IL)-1β may be a pro-metastatic cytokine. However, we have not yet noted the clinical association between tumoral expression or serum level of IL-1β and RCC in our patient cohort. Herein, we investigate molecular mechanisms elicited by IL-1β in RCC. We found that IL-1β stimulates substantial monocyte chemoattractant protein (MCP)-1 production in RCC cells by activating NF-kB and AP-1. In our xenograft RCC model, intra-tumoral MCP-1 injection down-regulated Ki67 expression and reduced tumor size. Microarray analysis revealed that MCP-1 treatment altered protein-folding processes in RCC cells. MCP-1-treated RCC cells and xenograft tumors expressed MCP-1-induced protein (MCPIP) and molecules involved in endoplasmic reticulum (ER) stress-mediated apoptosis, namely C/EBP Homologous Protein (CHOP), protein kinase-like ER kinase (PERK), and calnexin (CNX). ER stress-mediated apoptosis in MCP-1-treated RCC cells was confirmed using Terminal deoxynucleotidyl transferase dUTP Nick-End Labeling (TUNEL) assay. Moreover, ectopic MCPIP expression increased PERK expression in Human embryonic kidney (HEK)293 cells. Our meta-analysis revealed that low MCP-1 levels reduce 1-year post-nephrectomy survival in patients with RCC. Immunohistochemistry indicated that in some RCC biopsy samples, the correlation between MCP-1 or MCPIP expression and tumor stages was inverse. Thus, MCP-1 and MCPIP potentially reduce the IL-1β-mediated oncogenic effect in RCC; our findings suggest that ER stress is a potential RCC treatment target.

## 1. Introduction

Renal cell carcinoma (RCC) is one of the 10 most common cancers in both men and women, with the third highest incidence among urological malignancies. The American Cancer Society predicted that in 2019, the United States will have approximately 73,820 new cases of RCC and approximately 14,770 related deaths. Clear cell RCC (ccRCC), the most common histological subtype of RCC, accounts for 75% of RCC cases [1]. Approximately 20–30% of patients with RCC present with local or distant metastasis at the time of diagnosis [2], which has a serious impact on disease-related mortality. Therefore, further research on the mechanisms implicated in RCC development and progression is highly warranted to identify new therapeutic targets.

A comprehensive genome-wide analysis highlighted the importance of genes participating in cellular metabolism, oxygen sensing, such as von Hippel–Lindau (VHL)/ Hypoxia-inducible factor (HIF) and PI3K/AKT pathways, as well as the maintenance of chromatin states, such as Polybromo (PBRM) I and SET domain-containing protein (SETD) 2, in ccRCC [3]. This implied that ccRCC development and progression may be driven by oncogenic–metabolic shifts and epigenetic reprogramming. In addition to genetic mutations and epigenetic aberrations, accumulating evidence indicates that inflammatory molecules play roles in RCC carcinogenesis. Systemic inflammation markers, such as increased levels of neutrophil and platelet counts and high neutrophil–lymphocyte ratios, are associated with poor prognosis of patients with advanced RCC [4]. Inflammatory cytokines, IL-6 and IL-8, are secreted by VHL-deficient RCC cells after exposure to hypoxia [5]. IL-6 induces the expression of suppressor of cytokine signaling-3 (SOCS3), one of the factors associated with aggressiveness of RCC. Furthermore, increased IL-6 levels may be responsible for drug resistance in interferon-treated RCC [6]. IL-8 can promote the epithelial–mesenchymal transition of RCC by activating the AKT signal transduction pathway [7]. According to a cell-based study [8], IL-1β, a crucial pro-inflammatory mediator, was reported to enhance metalloproteinase-dependent invasion of RCC cells through the activation of CCAAT enhancer–binding protein β (CEBPB). In addition to these inflammatory cytokines, the importance of monocyte chemoattractant protein-1 (MCP-1; also known as chemokine (C–C motif) ligand 2, CCL2), a key mediator of monocyte recruitment, in RCC is well documented. Wang’s study [9] reported that both high levels of MCP-1 and its receptor, CCR2, expression were correlated with poor prognosis of patients with non-metastatic ccRCC. Arakaki’s study [10] shown that MCP-1 expression correlated with tumor growth, angiogenesis, and macrophage infiltration in a xenograft model of RCC. Moreover, Arakaki’s work showed that macrophage depletion from RCC xenografts overexpressing MCP-1 markedly suppressed tumor growth and angiogenesis, suggesting that MCP-1 inhibition exhibits anti-tumor effects against RCCs, at least partially, through the reduction of macrophage recruitment into the tumor [10]. As the immune system and inflammatory response are very complicated in nature, any study limited to a single immune molecule will yield fragmented results and being invalid for clinical application [11]. Despite IL-1β and MCP-1 have reported to be oncogenic [8,9,10], their potential anti-tumor activities cannot be excluded. IL-1β can induce a variety of downstream effectors through IL-1β/IL-1R signaling, depending on cellular microenvironment and cell types [12,13,14]. The downstream effectors then can modulate the effect of IL-1β. Similarly, the role of MCP-1 can be more complex in RCC. In addition to monocyte recruitment, studies have shown that MCP-1 causes cell death by induction of MCP-1-induced protein (MCPIP)-1 [15,16]. MCPIP-1 possesses transcriptional activity and its expression induces genes involved in ER stress and apoptosis in both human embryonic kidney HEK 293 cells and the cardiomyoblast cells. These findings suggest that despite the oncogenicity caused by macrophage recruitment, MCP-1 may induce RCC cell apoptosis and therefore exert an anti-cancer effect.

Although IL-1β is assumed to be a pro-metastasis cytokine in RCC, supporting clinical evidence is still lacking. To fill the gap, herein, we examined clinical samples, including tumor and matched non-tumor biopsies and sera, collected from pretreated patients with ccRCC and investigated the role of IL-1β and complex molecule contexture elicited by IL-1β in ccRCC.

## 2. Results

### 2.1. No Significant Association Between IL-1β and Clinical Consequences in Patients with RCC

**Figure 1:**
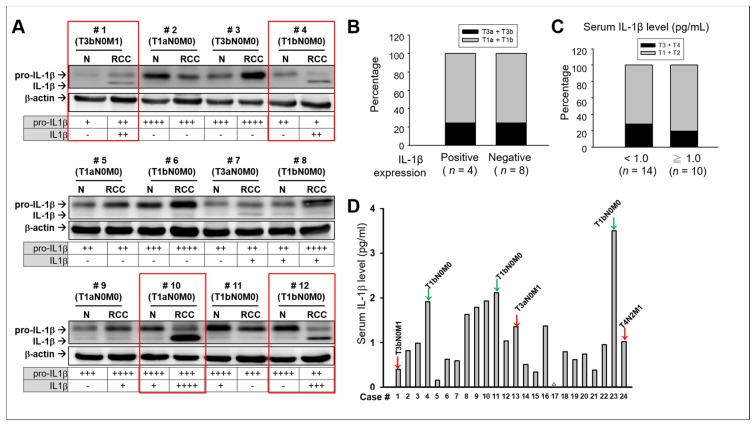
Intra-tumoral expression patterns and serum levels of IL-1β in RCC. (**A**) Western blot analysis of pro-IL-1β and IL-1β in human RCC tumor tissues (RCC) and the adjacent normal renal tissues (N). The protein bands of pro- IL-1β (31 kDa) and IL-1β (17 kDa) were quantified using ImageJ and normalized with the amounts of β-actin. The normalized intensities were graded into four classes, high (+++), medium (++), low (+), and negative (–). Red boxes indicate IL-1β-positive (highly and moderately stained) RCC. (**B**) Statistical analysis for determining the clinical stage of patients in the IL-1β-positive group compared with that of patients in the IL-1β-negative group. (**C**) Statistical analysis for determining the clinical stage of patients in the high (≥1.0 pg/mL) serum IL-1β group compared with that of patients in the low (<1.0 pg/mL) serum IL-1β group. Statistical analysis was performed using Mann–Whitney nonparametric *U*-test. (**D**) The serum levels of IL-1β were determined using the enzyme-linked immunosorbent assay (ELISA). △ indicates un-detectable. Red arrows indicate that the RCC diagnosis was metastasis (M1 and/or N1) and/or late stage; green arrows indicate that neither was diagnosed.

Functionally active IL-1β (17.5 kDa) is produced only after the inflammasome-induced proteolytic cleavage of pro-IL-1β (31 kDa). Discriminating between IL-1β and pro-IL-1β through immunohistochemical (IHC) analysis is difficult because no IHC antibody exists that is specific to IL-1β but not to pro-IL-1β. Hence, instead of performing IHC analysis, we performed Western blotting analysis, which can efficiently separate pro-IL-1β and IL-1β based on their molecular weights. Paired tumor and adjacent non-tumor tissues were collected from 12 patients with RCC. On the basis of results from Western blotting, we classified these RCC samples into two groups, the IL-1β-positive (highly and moderately stained, red boxes, *n* = 4) and the IL-1β-negative (not stained and slightly stained, *n* = 8) (Figure 1A). We did not observe an association between the intra-tumoral IL-1β expression and clinical stage of patients with RCC (Figure 1B). The percentage of patients at the late stages of cancer (T3a and T3b) was the same in both groups. Case 1, showing significant intra-tumoral IL-1β, was the only case that had a diagnosis of distant metastasis and late-stage cancer; cases 3 and 7, diagnosed with late-stage RCC tumor, had none and trace (+) amount of IL-1β, respectively. Furthermore, case 10, which showed the strongest staining for IL-1β, was diagnosed as early stage without distant metastasis or lymph node metastasis. Moreover, we measured the serum levels of IL-1β and classified patients into two groups, groups with high (>1.0 pg/mL, *n* = 10) and low (<1.0 pg/mL, *n* = 14) serum IL-1β (Figure 1C). More patients (4/14; 28.6%) with late stages of cancer were in the low IL-1β-serum-level group than those (2/10, 20%) in the high IL-1β-serum-level group (Figure 1D). Although no statistically significant differences were observed due to the limited number of samples, probably RCCs with aggressive phenotypes (red arrows) did not demonstrate extremely high serum IL-1β levels, whereas patients with high serum IL-1β levels, such as cases 4, 11, and 23 (green arrows), were not diagnosed as having aggressive RCC. These results suggest that neither high intra-tumoral levels nor serum IL-1β levels necessarily indicate poor prognostic RCC or vice versa. Moreover, in some cases, high intra-tumoral (case 10) or serum levels of IL-1β (case 23) were observed in patients with good clinical performance. It was possible that IL-1β elicits downstream molecules possessing anti-tumor activities to modulate its function in RCC.

### 2.2. IL-1β Induces MCP-1/ MCPIP-1 Signaling in RCC

**Figure 2:**
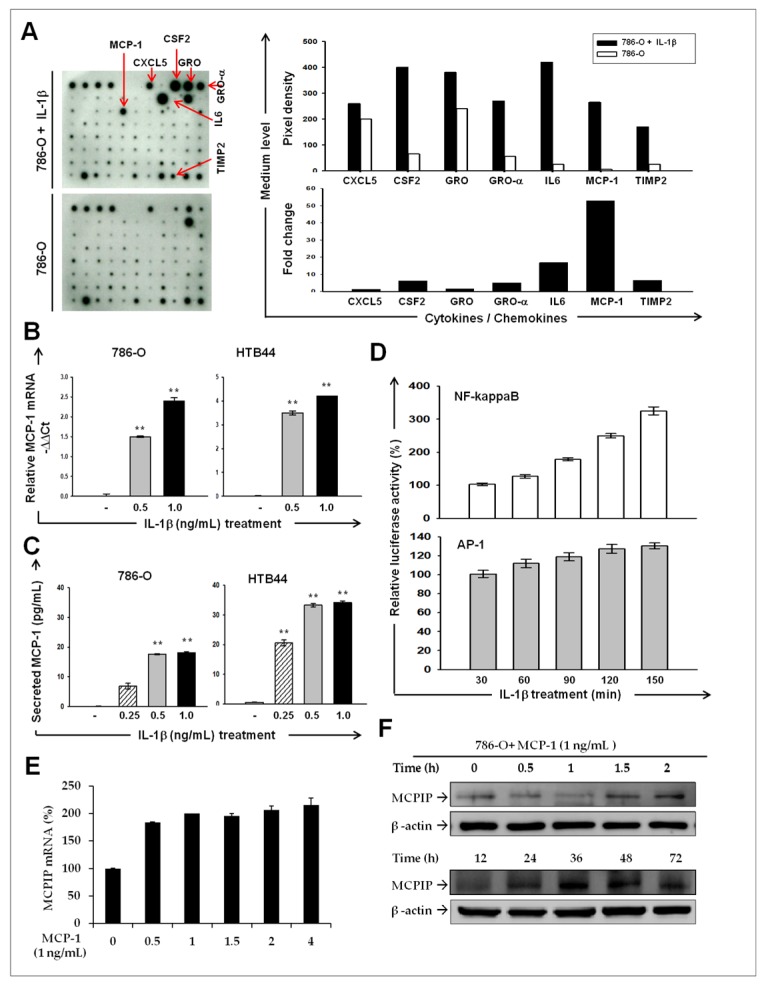
IL-1β induced monocyte chemoattractant protein (MCP)-1/ MCP-induced protein (MCPIP)-1 signaling pathway in RCC. (**A**) Human cytokine antibody arrays were used to detect secreted cytokines in the media of the 786-O cells after stimulation with IL-1β for 24 h. Left panels: the original images of the hybridized cytokine array (left). Red arrows indicated spots with significant increased or decreased in the IL-1β-treated cells. Intensities of spots were quantified using ImageJ and shown as pixel densities (upper) or as fold changes (lower) relative to the untreated cells. IL-1β stimulates MCP-1 mRNA expression (**B**) and protein secretion (**C**) by renal cell carcinoma (RCC) cell lines in a dose dependent manner. Cells were treated with indicated concentrations of IL-1β for 24 h followed by quantitative PCR or ELISA to determine the MCP-1 mRNA expression and secretion, respectively. Results are expressed as mean ± SD (*n* = 3). ** *p* < 0.01 versus phosphate-buffered saline (PBS) controls. (**D**) IL-1β induced the transcriptional activities of NF-κB and activator protein (AP)-1. Luciferase reporter assays for NF-κB- and AP-1 were carried out at indicated times after IL-1β treatment. The luciferase activity determined and normalized to total protein (mean ± SD from three independent tests. ** *p <* 0.01 versus control. Time-course quantitative RT-PCR (**E**) and Western blotting analysis (**F**) were carried out to examine the mRNA or protein expression of MCPIP, respectively. β-actin was used as an internal control.

To identify the IL-1β-induced downstream mediators in RCC, we performed cytokine antibody array analysis by using the conditioned medium of 786-O cells with or without IL-1β. In response to IL-1β stimulation, the conditioned medium of 786-O cells contained significantly increased levels of C-X-C motif chemokine ligand (CXCL)5, colony stimulating factor (CSF), growth-regulated oncogene (GRO)-α, IL-6, monocyte chemoattractant protein (MCP)-1, and tissue inhibitors of metalloproteinase (TIMP) 2. Among these, the increase in MCP-1 levels on IL-1β induction had increased up to 53 times compared with untreated controls (Figure 2A). Moreover, quantitative PCR and ELISA indicated substantial increases in mRNA expression (Figure 2B) and secretion (Figure 2C) of MCP-1 in response to IL-1β treatment in both 786-O and HTB44 cells, confirming that MCP-1 production is induced by IL-1β. Luciferase reporter assays also indicated that the transcriptional activities of both nuclear factor kappa B (NF-κB) and activator protein (AP)-1, key transcriptional factors regulating MCP-1 expression, considerably increased up to 3.2 and 1.3 fold over time during IL-1β treatment for 150 min (Figure 2D). It has been reported that MCP-1 induces MCPIP-1 expression to elicit ER stress-induced apoptosis [15,16]. Because RCC cells express chemokine (C–C motif) receptor type (CCR) 2, the cell membrane receptor of MCP-1 [17], we examined whether MCPIP-1 levels increase after MCP-1 treatment in 786-O cells. As shown in Figure 2E, MCPIP-1 mRNA expression markedly elevated at 0.5 h of 5 ng/mL MCP-1 treatment and remained at a high level. Western blotting analysis demonstrated that MCPIP-1 protein levels decreased at 0.5 and 1 h and then substantially increased at 1.5 h and beyond, reaching a peak at 36 h of MCP-1 treatment. (Figure 2F). These results indicate that IL-1β induces the production of MCP-1; MCP-1 then enhances MCPIP-1 expression in RCC cells in an autocrine manner. The results also suggest that the MCP-1/MCPIP-1 signaling pathway may be established in RCC cells in response to elevated levels of IL-1β.

### 2.3. Treatment of MCP-1 Resulted in Dysregulation of Protein-Folding and Expression of ER Stress Mediators in RCC Cell Line

**Figure 3:**
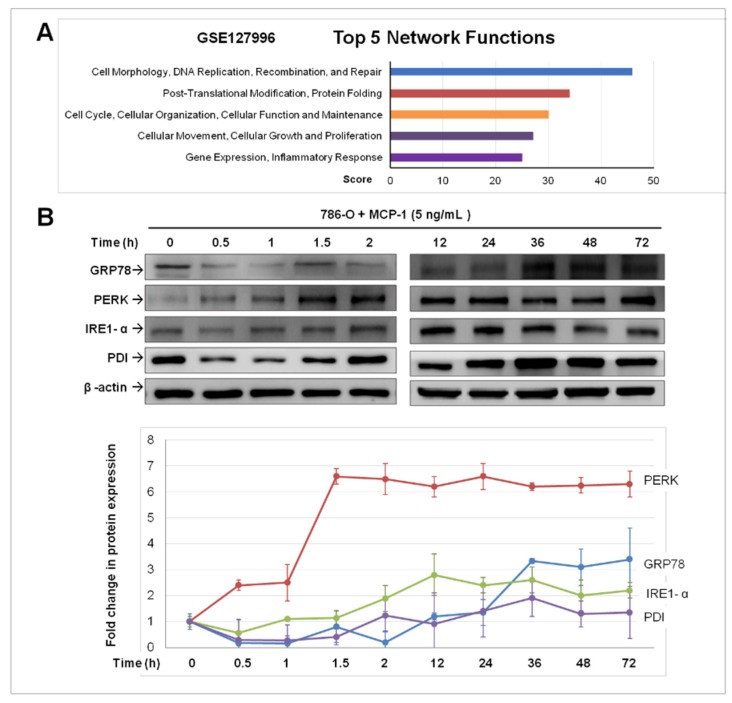
Dysregulation of protein-folding and expression of endoplasmic reticulum (ER) stress mediators in 786-O cells by monocyte chemoattractant protein (MCP)-1 treatment. (**A**) A graph showing the top 5 network functions changed in MCP-1-treated 786-O cells. (**B**) Western blotting analysis of indicated protein markers of ER stress in response to MCP-1 treatment in 786-O cells. After MCP-1 (5 ng/mL) treatment of 786-O cells, whole cell lysate was harvested at the indicated time points. β-actin was used as a loading control. Densitometric analysis of immunoblots for indicated proteins in untreated (0 h) and MCP-1-treated 786-O cells. The level of each protein in untreated control was set as 100%. Data is represented as mean ± SD (*n* = 3).

To identify the biological process affected by MCP-1/MCPIP-1 signaling, we performed gene expression microarray analysis for 786-O cells with and without MCP-1 treatment for 24 h. Ingenuity pathway analysis was used to classify differentially expressed genes based on molecular function; the results revealed that the group of genes involved in protein-folding was ranked the second of the differentially expressed genes in MCP-1 treated cells (Figure 3A). The unfolded protein response (UPR) is a cellular stress response related to the endoplasmic reticulum (ER) stress. We then examined whether MCP-1 induced expression of ER stress sensors/markers, including binding immunoglobulin protein (BiP)/glucose-regulated protein (GRP) 78, RNA-dependent protein kinase-like ER kinase (PERK), inositol requiring element (IRE)1-α, and protein disulfide isomerase (PDI). As shown in Figure 3B, GRP78 did not increase (and even decreased within 2 h) after MCP-1 treatment. However, GRP78 began to increase at 36 h and plateaued in the remaining experimental period. MCP-1 treatment rapidly and markedly increased PERK expression, which peaked at 1.5 h and was at consistently high levels throughout the experimental period. Initially, the expression of IRE1-α and PDI decreased slightly at 1.5 h of MCP-1 treatment. From 2 h onwards, PDI expression increased steadily. Similarly, IRE1-α had a significant increase at 2 h, and its up-regulation was maintained in the remaining experimental period. These data suggested MCP-1 may act as downstream effectors of IL-1β, not only induced MCPIP-1 expression, but also directly or indirectly led to ER stress in RCC cells.

### 2.4. ER Stress-Mediated Apoptosis Occurred in MCP-1-Treated Cultured 786-O Cells

**Figure 4:**
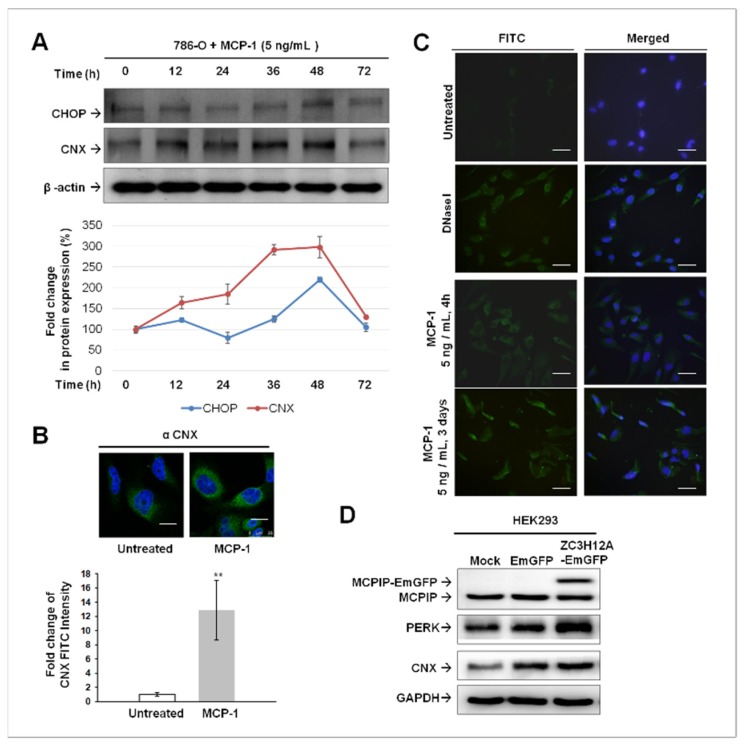
Induction of ER stress-mediated apoptosis by MCP-1/MCPIP-1 signaling. (**A**) Western blotting analysis of CHOP and CNX expression in MCP-1 treated 786-O cells. After MCP-1 (5 ng/mL) treatment of 786-O cells, whole cell lysate was harvested at the indicated time points. β-actin was used as a loading control. (**B**) Cellular immunofluorescence staining for calnexin [CNX, Fluorescein isothiocyanate (FITC) labeled] in 786-O cells with or without treatment of MCP-1 for 16 h. Cells were visualized under a fluorescent microscope (Leica TCS SP8 X, Leica microsystems) and FITC intensity was quantitated using an image analyzer system Leica application Suite X (LAS X). The bars represent means ± SD (*n* = 3). ** *p* < 0.01 versus untreated controls. Scale bar, 10 µm. (**C**) TUNEL assay to determine MCP-1-induced apoptosis of 786-O cells. Cells were treated with MCP-1 (5 ng/mL) for 4 h or 3 days and induction of apoptosis was confirmed by the appearance of TUNEL-positive cells. DNase I treated (10 U/mL for 30 min) cells were used as a positive control. Scale bar, 50 µm. (**D**) Cellular protein lysate was harvested at 24 h after transfection with plasmids encoding either EmGFP or MCIPIP-EmGFP. Protein expression of MCPIP, PERK, and CNX were analyzed by immunoblotting analysis. Glyceraldehyde-3-Phosphate Dehydrogenase (GAPDH) was used as loading controls for immunoblotting analysis.

If ER stress is prolonged and the adaptive UPR fails, apoptotic cell death ensues. PERK was reported as an essential component of ER-mitochondrial contact sites and crucial for conveying apoptosis after ROS-based ER stress [18]. In addition, CCAAT enhancer–binding protein homologous protein (CHOP) [19,20] and calnexin (CNX) [20,21,22,23] play important roles in ER stress-induced apoptosis. We next investigated whether ER stress-mediated apoptosis involved in the events induced by MCP-1/MCPIP-1 signaling in RCC cells by analyzing the expression of CHOP and CNX. As illustrated in Figure 4A, long-term exposure to MCP-1 led to an increased expression of CHOP and CNX, which reached their peaks at 48 h and which then decreased at 72 h. Through cell immunofluorescence staining, we confirmed CNX induction by MCP-1 in 786-O cells (Figure 4B). We next performed a TUNEL assay to examine the apoptotic cell death in MCP-1 treated 786-O cells. MCP-1 (5 ng/mL) rapidly induced apoptosis after 4 h of treatment; this effect was maintained until the end of the treatment period (3 days) (Figure 4C). Altogether, these data indicated ER stress-mediated apoptosis occurred in response to treatment of MCP-1. To examine whether that MCPIP-1 was involved in PERK or CNX induction by MCP-1, a human embryonic kidney cell line HEK293 was transfected with plasmids expressing either MCPIP-1-Emerald Green Fluorescent Protein (EmGFP) (ZC3H12A-EmGFP) or EmGFP. As shown in Figure 4D, the endogenous MCPIP-1, PERK, and CNX were present in HEK293 cells with or without transfection. The amount of PERK protein apparently increased along with ectopic MCPIP-1-EmGFP expression at 24 h after transfection. CNX levels slightly increased in MCPIP-EmGFP-expressing cells compared with EmGFP-expressing cells. These data suggest that MCP-1/ MCPIP-1 signaling may sensitize cells to apoptosis under prolonged ER stress through inducing, at least, PERK expression. 

### 2.5. Intra-Tumoral Injection of MCP-1 Led to Expression of MCPIP-1, PERK, and CNX, as well as Reduced Tumor Size in a Mouse Xenograft Model of RCC

**Figure 5:**
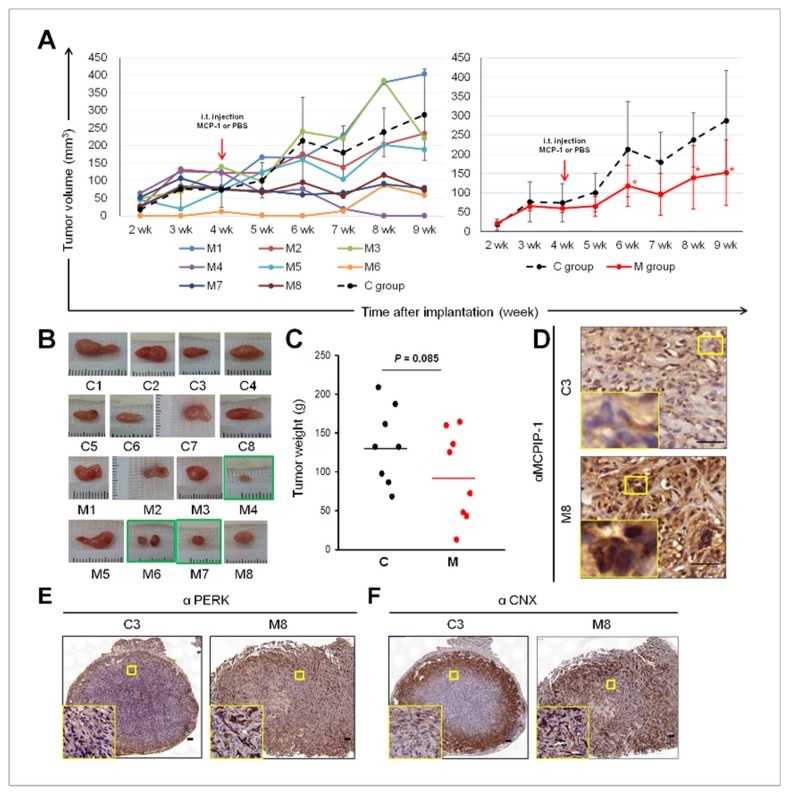
Tumor suppressing effect of MCP-1 in a mouse xenograft model of RCC. (**A**) Growth curve for individual (left chart, solid lines) and average (right chart, the red solid line) tumor volume upon treatment of MCP-1 (M group) were presented. The black dash lines expressed as the average tumor volume of the C group. Tumor volumes were monitored once a week and calculated as described in the “Materials and methods”. Photograph (**B**) and weight (**C**) of xenograft RCC tumors on Day 63. Representative images of MCPIP-1 (**D**), PERK (**E**), and CNX (**F**) immunohistochemical detection of in 786-O xenograft tumor samples from C and M group BALB/C nude mice. The staining was visualized and photographed on an inverted microscope (Nikon, Eclipse TE300, Tokyo, Japan). Scale bar, 50 µm. Yellow squares, magnitude x 200.

We designed a mouse xenograft model of RCC through the i.t. injection of MCP-1 (0.75 µg/mouse) weekly starting from 4 weeks (M group, *n* = 8) after 786-O cell implantation. The mice treated through the i.t. injection of the same volume of PBS were use as controls (C group, *n* = 8). For tumor size data over days 7–63 after 786-O implantation, most tumors of M group mice exhibited decreased tumor growth rate (Figure 5a, left chart) and a significant decrease in average tumor volume at week 6, 8, and 9 (Figure 5A, right chart) compared with the C group. Notably, from week 5, M4 tumors evidently shrank in size and M7 and M8 tumors almost stopped growing (Figure 5A, right chart), and resulted in considerable decreases in tumor weight at day 63 (green boxes in Figure 5B); the reduction in the average tumor weights of M groups did not reach statistical significance (Figure 5C). IHC analysis of tumor biopsies isolated from the RCC xenograft model revealed increased MCPIP-1 protein expression in tumors of M group, but it was absent or down-regulated in C tumors (Figure 5D). IHC also indicated that although the periphery of 786-O xenograft tumors from C and M groups demonstrated high staining intensity for PERK and CNX, the inner parts were strongly stained only in the M tumors (Figure 5E,F, respectively). These data indicated that the i.t. injection of MCP-1 had an induction effect on the expression of MCPIP-1, PERK, and CNX, and this may account for, at least partly, reduced size and weight of some xenograft RCC tumors in M group.

### 2.6. Prognostic Value of MCP-1 and MCPIP Expression in RCC

**Figure 6:**
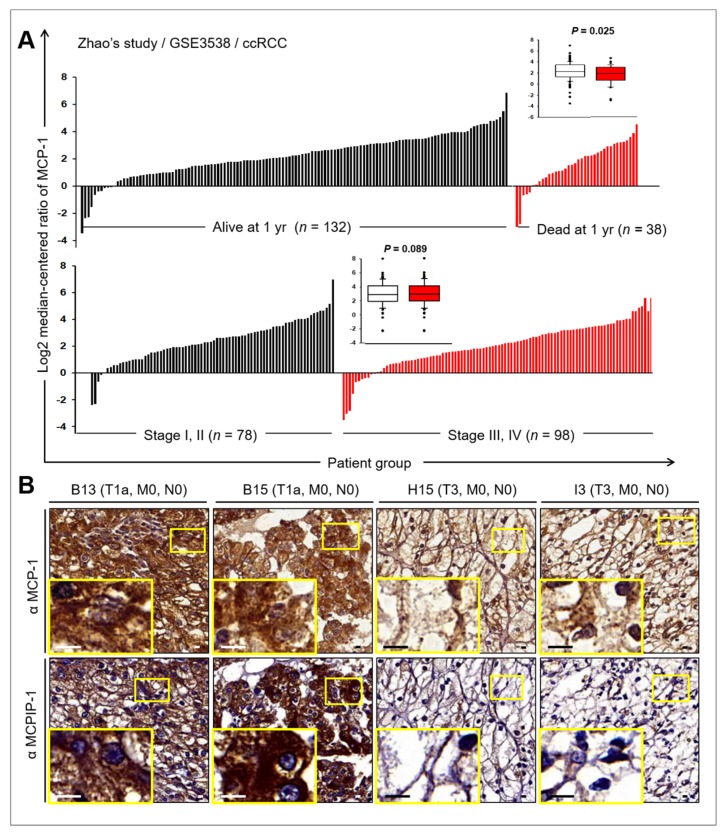
Expressions and prognostic values of MCP-1 and MCPIP in ccRCC. (**A**) Expression of MCP-1 mRNA in tumors of patients with ccRCC. The MCP-1 expression profiles of ccRCC with information about pathological characteristics were obtained from Zhao’s study [24]. Each of the tumor samples is plotted in order of increasing levels of MCP-1 mRNA. Inset: box plots display the median values of the array data and the 25th and 75th percentiles. The minimum and maximum values are indicated as whiskers. Points indicate outliers. Statistical differences were determined by Student *t*-test. (**B**) Immunohistochemical (IHC) staining of human ccRCC TMAs with anti-MCP-1 or anti-MCPIP antibodies. Representative images of immunohistochemistry for MCP-1 (upper panels) and MCPIP (bottom panels) in ccRCC tumors with pathological characteristics. Scale bar, 10 µm.

We surveyed publicly available microarray studies examining MCP-1 or MCPIP-1 expression in ccRCC in the Oncomine database (www.oncomine.org). Zhao’s array data [24] showed that more ccRCC patients with high MCP-1 expression (*n* = 132) survive than those who with low MCP-1 expression (*n* = 38) at 1 year after nephrectomy (*p* = 0.025, *t*-test; Figure 6A). On stratifying the patients according to the TNM Classification of Malignant Tumors (TNM) stages, MCP-1 expression was found to be higher in the early stages (T1 and T2, *n* = 78, signal log ratio = 2.38) than in the late stages (T3 and T4, *n* = 98, signal log ratio = 1.98), but it was not statistically significant (*p* = 0.098, *t*-test). Furthermore, we classified patients into two groups based on MCP-1 expression. The groups with high and low MCP-1 expression were not significantly different in tumor grades (Supplementary Figure S1). We used IHC to examine the presence of MCP-1 or MCPIP-1 protein in ccRCC tumor and adjacent non-neoplastic tissues in a commercial human ccRCC tissue microarray (TMA). Supplementary Table S1 lists the clinicopathological features of ccRCC in 75 patients included in the TMA. Cytoplasmic MCP-1 was detected in all samples and its expression in ccRCC tumors (median = 2, range = 1–3) was significant lower that in adjacent non-tumor tissues (median = 3, range = 2–3, *p* < 0.01; Table 1). No significant difference was found between ccRCC tumors (median = 1, range = 0–3) and adjacent non-tumor tissue (median = 1, range = 0–2; *p* = 0.30) for MCPIP (Table 1). Notably, more adjacent non-tumor tissues (65, 86.7%) were positive for MCPIP-1 than were tumor tissues (58, 77.3%; Table 1). MCP-1 and MCPIP-1 expression in the ccRCC at TNM stages was compared using the chi-square test. No significant associations were observed between MCP-1 or MCPIP-1 expression and ccRCC at TNM stages. However, twenty-four (24/62, 38.7%) tumors at an earlier stage (T1 and T2, *n* = 62) showed stronger staining for both MCP-1 and MCPIP-1, whereas five (5/13, 38.5%) aggressive (T3 and T4, *n* = 13) ccRCC tumors showed lighter staining for MCP-1 and barely detectable staining for MCPIP-1 (Figure 6B). Additional file 1: Table S2 lists the results of statistical analysis regarding TNM stage and MCP-1 and MCPIP-1 expression. Because of the limited sample size, we could not assess the association of tumor invasion with MCP-1 and MCPIP-1 expression.

## 3. Discussion

Biomarker discovery through bench work is typically challenged by clinical validation [25]. Although IL-1β reportedly mediates RCC invasion by inducing matrix metallopeptidases (MMPs) expression in 786-O cell line [8], at present, it is still lack of supporting clinical evidence. We found no association between IL-1β and tumor aggressiveness in our RCC biopsies. Instead, some cases with elevated levels of intra-tumoral or serum IL-1β exhibited early T stages and non-metastatic. We noted that IL-1β stimulated substantial MCP-1 secretion in RCC. Sequentially, MCP-1 induced the expression of MCPIP-1. Both MCP-1 and its receptor CCR2 are involved in various diseases. The role of theMCP-1/CCR2 axis in oncology can be complex. Despite oncogenic role of MCP-1 was reported [9,10,17], pancreatic cancer patients with high circulating levels of MCP-1 demonstrated significantly higher survival rate than do those with low MCP-1 levels [26]. Monocytes attracted by MCP-1 exert direct anti-proliferative and pro-apoptotic activities toward pancreatic cancer cells; this accounts for anti-malignant activity of MCP-1 in pancreatic cancer [26]. In RCC, the blockage of MCP-1/CCR2 signaling inhibits cell proliferation, but MCP-1 treatment does not affect cell growth [10,17]. Research on heart failure has discovered that MCP-1 causes cell death by inducing MCPIP-1 [15,16]. MCPIP-1 has transcriptional activity, and its expression induces the expression of genes involved in oxidative and ER stress as well as apoptosis in both HEK 293 cells and the cardiomyoblast cells H9c2. Our data showed that MCP-1 treatment not only induced the expression of MCPIP-1 but also markers of ER stress. Notably, proteins being important for ER stress-induced apoptosis, such as PERK, CHOP, and CNX were also induced by MCP-1 or MCPIP-1 in RCC cells and RCC xenograft tumors. These results suggest that RCC cells endure prolonged ER stress caused by metabolic dysregulation. Moreover, the activated MCP-1/MCPIP-1 signaling pathway enhances the sensitivity of RCC cells to ERstress-induced apoptosis by inducing PERK, CHOP, and CNX expression.

Growing evidence suggests that metabolic syndrome is strongly associated with incidence and progression of RCC [27,28]. Since metabolic disorder induces oxidative and ER stress [29], it can thus be inferred that RCC may endure long-term oxidative and ER stress. A 37-year–follow-up epidemiological study identified that type 2 diabetes was an independent risk factor for RCC in women [30]. In addition, ccRCC is characterized by the accumulation of sterol in tumor cells, implying that dysregulation in lipid metabolism is crucial in RCC development and progression. A prospective study confirmed this assumption and found that elevated serum triglyceride levels were significantly associated with RCC [31]. Metabolic disorders caused by fatty acid (FA) and glucose exert cytotoxicity via their action on ER, eventually leading to ER stress [29]. Reactive oxygen species (ROS) are generated as cellular metabolism byproducts from FA and glucose in the mitochondria. Therefore, accumulating FA leads to excessive ROS; in turn, ROS interferes with the redox status of ER lumen and thus inhibit protein-folding as well as induce ER stress and the UPR. In addition to protein synthesis and folding, ER has a major role in lipid production, calcium storage, and homeostasis regulation [32]. There are several adaptive responses to maintain ER homeostasis, such as the UPR, ER-associated degradation, and autophagy [32,33]. In mammalian cells, the UPR is regulated by three transmembrane mediators, namely PERK, IRE1α, and activating transcription factor 6 (ATF6). Under normal conditions, these proteins are associated with GRP78 and remain inactive. Under ER stress, GRP78 dissociates from these sensor proteins and switches on the UPR cascade through PERK and IRE1α dimerization and autophosphorylation and ATF6 proteolysis. The consequences of UPR activation is inflammation, and on prolonged stress, apoptosis is induced. The relationship between metabolic disorders and ER stress may explain chronic inflammation in RCC [4]. This also implies that ER stress-induced apoptosis can be exploited to eliminate RCC cells. Mitochondria-associated ER membranes (MAMs) are microdomains where ER and mitochondria connect. Through MAMs, ER and mitochondria function together as sensors of nutritional imbalance and integrators of responses to metabolic stress [29]. The rapid transfer of calcium between ER and mitochondria through MAMs is crucial for cell signaling, adaption, and survival [29]. ER–mitochondria coupling through MAM promotes mitochondria respiration during the early phase of ER stress but reduces mitochondrial metabolism under sustained stress due to calcium overload–induced apoptosis [34,35]. Aberrations in either the composition or number of MAMs lead to an alteration in inter-organellar calcium dynamics and causes increased oxidative stress and metabolic disturbance. MAM is not only important in metabolic regulation but also key in cell death sensitivity. Verfaillie demonstrated that PERK, a key ER stress sensor of the UPR, is uniquely enriched in MAMs [18]. Their data indicated that PERK is required to establish ER–mitochondria juxtapositioning and convey apoptosis signals after ROS-based ER stress induction; thus, PERK loss may impair cell death sensitivity in pathological conditions linked to ROS-mediated ER stress [18]. CNX expression may similarly increase the sensitivity toward ER stress-induced apoptosis. Delom showed that CNX expression directly influenced the sensitivity of tunicamycin-induced apoptosis in breast carcinoma MCF-7 cells [21]. By binding to Bap31, CNX transformed the MCF-7 cell line from being tunicamycin-resistant to tunicamycin-sensitive. Along with MCPIP-1 induction, the up-regulation of PERK and CNX in RCC cells and xenograft tumors by MCP-1 treatment may sensitize RCC cells to ER stress-induced apoptosis by facilitating the propagation of ROS signals through enhancing MAMs and by forming Bap31-containing pro-apoptosis complex, respectively. This may explain tumor shrinkage caused by the intra-tumoral injection of MCP-1 and apoptosis observed in MCP-1-treated RCC cells. Moreover, we observed that the ectopic expression of MCPIP-1 significantly increased the protein amounts of PERK, thus indicating that MCPIP-1 is key to the PERK induction effect of MCP-1. Taken together, the MCP-1/MCPIP-1 signaling, indirectly established by IL-1β stimulation, may enhance ER stress-induced apoptosis sensitivity in RCC cells through the induction of PERK and CNX expression.

## 4. Materials and Methods

### 4.1. Patients and Clinical Samples

The clinical characteristics of ccRCC patients between 2011 and 2014 were reviewed using electronic medical records. The use of human specimens was approved by the Institutional Review Board of Chang Gung Memorial Hospital (Keelung, Taiwan, number: 104-7287B, 1 Jan 2016 to 31 Dec 2018). Informed consent was obtained from each patient. Nephrectomy or blood samples for 24 patients with ccRCC were collected at the Department of Nephrology, Chang Gung Memorial Hospital (Keelung, Taiwan) according to the standardized ISO/IEC 17025:2005 protocol. The pathological features of ccRCC patients were listed in Supplementary Table S3. Neoplastic and non-neoplastic area were selected from each sample and examined by paraffin section procedure to confirm the presence or absence of the tumor. One neoplastic and one non-neoplastic tissue from each nephrectomy were snap-frozen in liquid nitrogen and stored at −80 °C until being used for evaluation of pro-IL-1β and IL-1β expression by Western blotting analysis. Serum was separated from peripheral blood samples that were collected by venipuncture (10 mL; BD Vacutainer glass serum tube) and stored at −80 °C in aliquots. Serum IL-1β levels were measured using ELISA (R&D, DLB50, Quantikine, Minneapolis, MN, USA) according to the manufacturer’s instruction. All samples were analyzed in duplicate.

### 4.2. Cell Lines and DNA Transfection

The human kidney cancer 786-O (CRL1932), HTB44, and HTB46 cell lines were purchased from Food Industry Research and Development Institute (Hsinchu City, Taiwan) and were maintained in complete growth medium as recommended by American Type Culture Collection (ATCC; Manassas, VA, USA). DNA transfection was performed with Lipofectamine 2000 (Thermo Fisher Scientific, MA, USA) following the manufacturer’s protocol.

### 4.3. Chemicals and Reagents

Recombinant human (rh) IL-1β and MCP-1 were purchased from R&D System (Minneapolis, MN, USA). Western blot was performed using antibodies against human IL-1β, MCP-1, GRP78, ERO1α (Cell Signaling Technology, Danvers, MA, USA), and CHOP (GeneTex, Irvine, CA, USA). Anti-CNX (Cell Signaling Tech.) was used for Western blotting, immunofluorescent staining, and IHC. Anti-MCPIP (GeneTex) and anti-PERK (Cell Signaling Tech.) were used for Western blotting and IHC. Tunicamycin was from Sigma-Aldrich (St. Louis, MO, USA)

### 4.4. Western Blot Analysis

Frozen samples of renal tissues were reduced to small pieces with a razor blade and homogenized on ice three times (20 sec each) by a Polytron blender (SONICS, Newtown, USA) in lysis buffer (Cell Signaling Tech.) supplemented with a protease-inhibitor cocktail (Merck, Kenilworth, USA). Tissue homogenates were centrifuged at 750× *g* for 10 min at 4 °C and the supernatant was assayed for total protein content and stored at −80 °C until used for Western blotting analysis. Protein lysate collection for cultured cells and Western blot analysis were performed as described previously [36,37].

### 4.5. ELISA

Cells were seeding in a 24-well culture plate (2 × 10^5^ cells/well) and grown in 2 mL of serum-free medium for 16 h. The supernatants were collected and the secreted MCP-1 was quantified using ELISA for human MCP-1 (R&D System) following the manufacturer’s protocol. Concentrations of measured MCP-1 were normalized to the cell number determined in parallel.

### 4.6. Cytokine Antibody Array Analysis

Human Cytokine Antibody Array 1 (RayBiotech, Peachtree Corners, GA, USA) was used to measure the secreted cytokines in conditioned medium of IL-1β treated 785-O cells following the manufacturer’s protocol. Conditioned medium subjected to cytokine assay were prepared from 10^5^ cells seeded onto 6-cm dish and cultured in 2 mL of serum-free medium with or without IL-1β (1 ng/mL) treatment for 16 h.

### 4.7. Luciferase Reporter Assay

Cells expressing luciferase under NF-κB or AP-1 promoter were generated by transducing lentivirus carrying NF-κB or AP-1-luciferase constructs (Cignal lenti reporter system; Qiagen, Germantown, MD, USA), following the manufactures’ instruction. Luciferase reporter assays were then carried out as described previously [36].

### 4.8. Quantitative Real-Time qPCR (qPCR) 

The synthesis of cDNA from total RNA was performed with M-MLV reverse transcriptase (Promega, Madison, WI, USA). The primers required were designed by the online tool at the Universal Probe Library Assay Design Center (Roche Applied Science, https://www.roche-applied-science.com) and shown in Supplementary Table S4. QPCR was performed with Fast Start Universal Probe Master Kit (Roche Applied Science, Penzberg, Germany). The cycling parameters began with 95 °C for 10 min, followed by 40 cycles of 95 °C for 15 sec and 60 °C for 60 sec, followed by a melting curve analysis.

### 4.9. Gene Expression Microarray

786-O cells maintained in RPMI-1640/10% FBS were treated with rhMCP-1 for 24 h in duplicate in 100-mm dishes. Extraction of total RNA, RNA quality evaluation, and cDNA microarray experiments were performed according to Affymetrix standard protocols by the microarray core laboratory at the National Health Research Institutes. The gene chips (Clariom S Assay HT06, Affymetrix Inc., Santa Clara, CA, USA) were scanned with an Affymetrix GeneChIP Scanner 3000 7G; and the CEL files generated were analyzed using Affymetrix Expression Console Software (v. 1.4), which normalizes array signals using Signal Space Transformation (SST) and a robust multiarray averaging (RMA) algorithm. Normalized data were analyzed using Transcriptome Analysis Console (TAC) 3.0 software (Affymetrix). A paired *t*-test was applied to identify differentially expressed transcript genes between sample pairs and probes, with *p*-values less than 0.05 and fold-change ≥2 declared significant. Microarray expression data are available at the U.S. National Center for Biotechnology Information Gene Expression Omnibus (GEO) database under accession number GSE127996.

### 4.10. Mouse Xenogaft Tumor Model

All animal experiments were approved by the Institutional Animal Care and Use Committee of Chang Gung Memorial Hospital (IACUC number: 2016060102, Jun 2016 to Dec 2019). Our animal center is an the Association for Assessment and Accreditation of Laboratory Animal Care (AAALAC) certified center and this study was performed in accordance with all the relevant guidelines and regulations. Mice were housed in a temperature and humidity-controlled room maintained on standard rodent chow with unrestricted access to water. 786-O cells (5 × 10^6^ per mouse) were mixed with equal volumes of Matrigel (#356237, Corning, Corning, NY, USA) and injected subcutaneous (s.c.) into the back area of male BALB/C (nu/nu) nude mice (*n* = 8 for each group) aged 8 weeks. Tumors were measured by a caliper and the volume was calculated using V = A × B^2^ × 0.5 (A, long diameter; B, short diameter). Tumors were harvested from euthanized mice 63 days post-injection, snap-frozen in liquid nitrogen, and stored in −80 °C. For IHC studies, formalin fixation followed by paraffin embedment was performed.

### 4.11. Imunohistochemistry (IHC) and Quantitative Staining Measurement of IHC

Xenograft tumors derived from 786-O cells were subjected to formalin fixation followed by paraffin embedment. Tissue sections (4 µm) were then prepared. IHC was performed with Dako REAL™ EnVision™ Detection System (Agilent, Santa Clara, CA, USA) according to the manufacturer’s instructions. Xenograft RCC tumor sections and human ccRCC tissue microarray (HKid-CRCC150CS-01, US Biomax, Inc.) were deparaffinized and dewaxed in xylene, and rehydrated through graded alcohol. Antigen retrieval was carried out with retrieval solution (Dako Target Retrieval Solution) at 95–100 °C for 10 min. Block endogenous peroxidase activity was performed by incubating sections in Peroxidase-Blocking Solution (Dako REAL™ Peroxidase-Blocking Solution) at room temperature for 10 min. After blocking non-specific binding with 1% BSA in PBS, tissue sections were incubated with blocking solution containing anti-MCPIP (mouse monoclonal antibody, GeneTex, USA, 1: 100), anti-PERK (mouse monoclonal antibody, Cell Signaling Technology, MA, USA, 1: 100), anti-CNX (mouse monoclonal antibody, Cell Signaling Technology, MA, USA, 1: 100), and anti-Ki67 (mouse monoclonal antibody, Abcam, 1: 100) at 4 °C for overnight. Followed by stained with Dako REAL™ EnVision™/HRP solution and Dako REAL™ DAB+ Chromogen: Dako REAL™ substrate solution. Counterstaining was performed with Hematoxylin solution. Finally, the slides were viewed and scanned using a Panoramic Digital Slide Scanner MIDI (3DHISTECH, Budapest, Hungary). The immunoreactivity for protein studies was assessed by pathologists. The grades of MCP-1 and MCPIP staining intensity at RCC tissue microarray were four-tiered as follows: 0, negative staining; 1, weak staining; 2, moderate staining; and 3, strong staining.

### 4.12. TUNEL Assay

Terminal deoxynucleotidyl transferase-mediated dUTP nick end labeling (TUNEL) was performed using Click-iT^®^ TUNEL Alexa Fluor Imaging Assay (Thermo Fisher Scientific, Waltham, MA, USA) to detect the apoptosis in the cells. Positive controls were obtained by the treatment of cells with DNase I, according to the manufacturer’s instructions. The samples were analyzed using a fluorescence microscope (Carl Zeiss International, Oberkochen, Germany).

### 4.13. Statistical Analysis

Comparisons of the results between various experimentally treated groups and their corresponding controls were carried out by Student’s *t*-test. Data presented are the means ± SD of *n* independent experiments as described in the Figures’ legends. Statistical significance was considered difference when *p* < 0.05 and significance when *p* < 0.01. The chi-square test performed with the computing environment R (v. 3.5.2., R Development Core Tam, 2018) and Mann–Whitney U test was used to assess the association between immunoreactivities of MCP-1 or MCPIP with TNM stages of ccRCC tumors. Statistical differences with *p* < 0.05 were considered significant.

## 5. Conclusions

We discovered that some RCC tumors with high levels of IL-1β were at the early stage and were non-metastatic. We demonstrated that IL-1β induces MCP-1/MCPIP-1 signaling in RCC cells. Consistent with MCPIP-1 induction, molecules involved in ER stress and ER stress-mediated apoptosis are up-regulated in MCP-1 treated RCC cells and xenograft RCC tumors. Apoptotic cell death was observed in MCP-1 treated RCC cells. In summary, our findings provide novel insights into the complex molecular contexture that is elicited by IL-1β in RCC, and they suggest that targeting ER stress is a possible therapeutic intervention for RCC.

## Figures and Tables

**Table 1:** Immunohistochemistry results for MCP-1 and MCPIP in ccRCC.

Protein Expression	No. of Cases	Percentage
MCP-1 in tumor tissue		
Negative	0	0
Positive	75	100%
Weak	30	40%
Moderate	29	38.7%
Strong	16	21.3%
MCP-1 in adjacent non-tumor tissue		
Negative	0	0
Positive	75	100%
Weak	7	9.3%
Moderate	33	44%
Strong	35	46.7%
MCPIP in tumor tissue		
Negative	17	22.7%
Positive	58	77.3%
Weak	21	28%
Moderate	32	42.7%
Strong	5	6.7%
MCPIP in adjacent non-tumor tissue		
Negative	10	13.3%
Positive	65	86.7%
Weak	40	53.3%
Moderate	25	33.3%
Strong	0	0

Monocyte chemoattractant protein (MCP)-1, MCP-induced protein (MCPIP), clear cell renal cell carcinoma (ccRCC).

## Supplementary Materials

Supplementary materials can be found at https://www.mdpi.com/1422-0067/20/23/6101/s1.

## Abbreviations

MCP-1	Monocyte hemoattractant protein-1
MCPIP-1	MCP-1-induced protein-1
IL-1β	Interleukin-1β
RCC	Renal cell carcinoma
BiP/GRP	Binding immunoglobulin protein/glucose-regulated protein 78
PERK	RNA-dependent protein kinase-like ER kinase
IREα	IRE: inositol requiring element 1α
PDI	Protein disulfide isomerase
ERO-1α	ER oxidoreductase 1α
CHOP	CCAAT enhancer–binding protein homologous protein
CNX	calnexin
SOCS3	Suppressor of cytokine signaling-3
NF-κB	Nuclear factor-κB
AP-1	Activator protein 1
ccRCC	Clear cell renal cell carcinoma
ER	Endoplasmic reticulum
TUNEL	Terminal deoxynucleotidyl transferase-mediated dUTP nick end labeling assay
IHC	Immunohistochemistry
VHL	von Hippel–Lindau
