# Supplementary material for: MCP-1/MCPIP-1 Signaling Modulates the Effects of IL-1β in Renal Cell Carcinoma through ER Stress-Mediated Apoptosis
Source: Int J Mol Sci. 2019 Dec 3;20(23):6101. doi: 10.3390/ijms20236101 (PMC6928829; doi:10.3390/ijms20236101)
Supplement: Supplementary file 1 [file ijms-20-06101-s001.pdf]

Supplementary Material

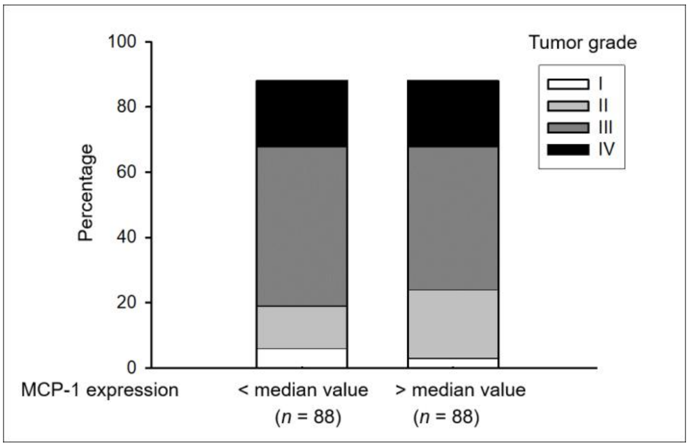

Supplementary Figure S1. Statistical analysis of Fuhrman grade of patients in the MCP-1-low expression group (< median value, n = 88) compared with patients in the MCP-1-high expression group (> median value, n = 88). Statistical analysis was performed using Mann-Whitney nonparametric U-test.

Table S1 Clinicopathological features of 75 patients in ccRCC tissue microarray

Variables	No. of cases	Percentage
Age (years)		
Mean	59	
Range	29-82	
Gender		
Male	100	67%
Female	50	33%
Pathological type		
ccRCC	75	50%
Non-tumor	75	50%
TNM stage		
I + II	62	83%
III + IV	13	17%
Lymph node metastasis		
Present	5	7%
Absent	69	93%
Distance metastasis		
Present	1	1%
Absent	74	99%

Supplementary Table S2 Comparison between early and late stage of ccRCC based on MCP-1 and MCPIP immunoreactivities using the chi-square test

	No. of cases	MCP-1 in tumor tissue		MCPIP in tumor tissue	
		IHC score	P-value	IHC score	P-value
TNM stage					
I	37	1.82 (0.76)		1.29 (0.89)	
II + III	38	1.86 (0.78)	0.992	1.52 (0.90)	0.987

Supplementary Table S3. Clinical pathologic characteristics of clear cell renal cell carcinoma patients.

Characteristics	No. of patients (*n* = 24)
Stage	
I	16
II	0
III	7
IV	1
TNM	
T4	1
T3a + T3b	7
T2	0
T1a + T1b	16
N1	2
N0	22
M1	3
M0	21

Supplementary Table S4 Primer sequences for qPCR

Primers for qPCR		
Gene symbol		Sequences
ZC3H12A	F	5' atcgatgggagcaacgtg 3'
	R	5' ccgctccagaaaccagttc 3'
b-actin	F	5' ccaaccgcgagaagatga 3'
	R	5' ccagaggcgtacagggatag3'
